# Vitamins and Celiac Disease: Beyond Vitamin D

**DOI:** 10.3390/metabo15020078

**Published:** 2025-01-28

**Authors:** Matteo Scarampi, Caterina Mengoli, Emanuela Miceli, Michele Di Stefano

**Affiliations:** 1st Department of Medicine, IRCCS “S.Matteo” Hospital Foundation, 27100 Pavia, Italy; matteo.scarampi@gmail.com (M.S.); c.mengoli@smatteo.pv.it (C.M.); e.miceli@smatteo.pv.it (E.M.)

**Keywords:** celiac disease, gluten-free diet, vitamin A deficiency, vitamin E deficiency, vitamin B12, vitamin B12 deficiency, folate deficiency, vitamin K deficiency

## Abstract

Celiac disease is a chronic inflammatory condition of the small bowel caused, in genetically predisposed subjects, by the ingestion of gluten and characterised by a broad clinical polymorphism, ranging from patients with an asymptomatic or paucisymptomatic disease. The clinical presentation ranges from the presence of minor, apparently unrelated symptoms or first-degree kinship with known patients to severe intestinal malabsorption and all its clinical consequences and complications. Even if a large body of research improved our understanding of the molecular basis of celiac disease pathophysiology, enhancing the identification of new targets for future new treatments, an accurate gluten-free diet remains the mainstay of the therapy for this condition, restoring a normal absorptive mucosa. It is very rare, nowadays, to deal with patients with severe malabsorption syndrome secondary to celiac disease. Consequently, physicians are currently less prone to search for nutritional deficiencies in celiac disease. To pinpoint the possibility of both a disease-related and a diet-induced vitamin deficiency, we reviewed the literature on vitamin deficiency in this condition and reported the impact both in untreated and treated patients with celiac disease. A gluten-free diet must be tailored for each patient to meet nutritional targets: the pre-existence or diet-induced intake inadequacies should be carefully considered for an effective management of celiac disease.

## 1. Introduction

Celiac disease (CD) is a chronic inflammatory condition of the small bowel caused in genetically predisposed subjects by the ingestion of gluten, a protein complex contained in wheat, rye and barley that is rich in glutamine and proline moieties [1,2]. The term “gluten” pertains to the whole protein component; on the contrary, when we use the term gliadin, we are considering the alcohol-soluble gluten fraction, containing a large part of the toxic component. Among others, the most characterized toxic peptide is a fragment of 33 amino acids of α-gliadin, which is resistant to gastric, pancreatic and intestinal degradation, crossing the intestinal mucosa barrier during the course of conditions and causing an increase of intestinal permeability that evokes an immunological response in the lamina propria.

CD is characterised by a broad clinical polymorphism, ranging from patients with an asymptomatic or paucisymptomatic disease who are diagnosed because of the presence of minor, apparently unrelated symptoms or first-degree kinship with known patients to subjects with a severe disease who suffer from a severe intestinal malabsorption and all its clinical consequences together with serious severe complications. On the contrary, intestinal lesions are always characterised by the presence of villous atrophy, which is associated with crypt hyperplasia and an increase in the intraepithelial lymphocyte count that configures a sort of flat mucosa [1,2].

CD is generally a benign condition: both histological alterations and clinical manifestations revert after the complete withdrawal of gluten from the diet. However, the frequent association with autoimmune disorders determines an increased morbidity [1,2], and both the higher risk of a T-cell clonal proliferations, predisposing the patient to enteropathy-type T-cell lymphoma, and the higher risk for neoplastic diseases, increase mortality [1,2].

Even if a large body of research improved our understanding of the molecular basis of the pathophysiology of this condition, enhancing the identification of new targets for future new treatments, an accurate and rigorous gluten-free diet (GFD) remains the mainstay of treatment. The removal from diet of the main causative factor allows the progressive extinguishing of the immunological response against gluten epitopes and the progressive improvement of intestinal lesions. Accordingly, a normal absorptive mucosa is restored and the required nutrient absorption is re-established [1,2].

During evolution, the nomadic life of humans permitted the procurement of foods by hunting or fishing and by fruit and vegetable collection. As humans were not yet involved in cultivation, we can infer that the Palaeolithic diet was obviously gluten-free. In south-western Asia, in particular, in an area including Iraq, Palestine, Syria, Lebanon and the south of Turkey (called the “Fertile Crescent”), the cultivation of some wild grains was started and humans begin to adapt their life to a nonmigratory model, preferring a resident life in villages. We can therefore infer that CD appeared after such a lifestyle and dietary modification occurred. Even if the first description of CD appeared in the 1st and 2nd centuries [3], the nosology of the disease dated back to the 19th century with its description in England and the United States [4,5]. The condition, first named Gee–Herter syndrome, was described in children as a severe malabsorption syndrome, causing diarrhoea, steatorrhoea and weight loss, and symptom onset was correlated to a modification of dietary habits, including the intake of gluten. For many years, CD was therefore considered a paediatric condition, severely symptomatic, causing an important impact on caloric intake and nutritional status and characterised by severe nutrient deficiencies. The first epidemiological studies were conducted in 1950, when typical malabsorption symptoms guided the diagnosis of CD, and a prevalence of 1:8000 in England and 1:4000 in Scotland were calculated [5]. The availability of a system for intestinal biopsy collection improved the epidemiology of CD, and the prevalence was set at 1:450 in Ireland, Scotland and Switzerland [6,7]. However, the availability of accurate serological tests revolutionised the history of CD epidemiology and prevalence figures of 1:99 and 1:106 were detected in Finnish [8] and Italian [9] schoolchildren, respectively. Similar rates of CD-associated serum autoantibodies were also positivity detected in adults in the UK and USA [10,11], with a prevalence figure of 1:87 and 1:105, respectively. In addition to the discussion of epidemiological issues, these studies provided also contain important information on clinical grounds: all these studies, in fact, showed unequivocally that a large part of CD patients were not yet diagnosed, due to the presence of an asymptomatic or pauci-symptomatic type of disease. Terms like “subclinical”, “minor” or “silent” referred to the clinical aspects of CD, in contraposition to “classical”, and began to circulate in the medical literature. The symbolic representation of “the CD iceberg” was depicted, stigmatizing the large number of patients in the submerged portion, while a minor number were located in the emerged portion of the allegoric iceberg [12,13,14].

Accordingly, in the last decades, CD has been more and more diagnosed, thanks to a radical change of the diagnostic paradigm: physicians developed an increased awareness of the importance of the relationship between CD and a group of associated conditions, rather than the presence of frank malabsorption symptoms [15]. The most important of these conditions are as follows: unexplained iron-deficiency (anaemia), autoimmune thyroiditis, type I diabetes, early-onset osteoporosis and, in particular, the familiarity. The chief consequences of this new paradigm are represented, first, by the evident reduction of diagnostic delay in the diagnosis of CD, second, by the earlier diagnosis of this condition in comparison with previous decades and third, by the recognition of less severe patients. This latter point is pivotal for nutritional issues. The large portion of newly diagnosed celiac patients are generally well-nourished patients, without severe nutritional deficiencies, in comparison with previously diagnosed patients, where micronutrient and vitamin deficiencies were very frequent. However, an extensive modification of dietary style, as a GFD implicates, may be associated with nutritional deficiencies if an experienced guide by dedicated personnel is not provided to patients; accordingly, a high risk of an insufficient nutritional intake of many micronutrients in both newly diagnosed patients or patients on a long-term GFD characterises this condition [16,17].

Although nowadays it is less frequent to diagnose CD patients with a severe malabsorption syndrome, it is important to underline the literature on nutritional deficiencies in this condition to refresh the possibility of both a disease-related and a diet-induced secondary vitamin deficiency. A GFD must be tailored to each patient to meet nutritional targets [16,17].

In a recent review paper [18], we described the alterations of serum vitamin D levels in patients with CD, stigmatizing the effect of the secondary hyperparathyroidism caused by alteration of the calcium balance on the seroconversion of 25(OH)-vitamin D to 1,25(OH)_2_ vitamin D. The effect of parathyroid hormone causes a reduction of serum 25(OH)-vitamin D and an increase of 1,25(OH)_2_ vitamin D levels: the lack of the determination of 1,25(OH)_2_ vitamin D serum levels, very unfrequently measured, may musk the real vitamin D status in CD patients, making evident the reduction of serum 25(OH)-vitamin D. This incorrect diagnostic approach may be responsible for the wrong prescription of vitamin D supplementation. Moreover, we also analysed serum vitamin D in treated and non-responder patients. Accordingly, in this review we focused on vitamin deficiencies other than vitamin D in celiac patients. In particular, we compared old papers with recent papers, underlining what should be considered as a consequence of the disease and what should be considered as a consequence of the gluten-free diet. The goal is to enhance the understanding of these clinical aspects and provide insights for future research in this field, contributing to improving the nutritional management of celiac patients.

## 2. Methods

### 2.1. Study Selection

A comprehensive literature search was conducted using PubMed to identify relevant studies dealing with the prevalence of vitamin deficiencies in untreated celiac disease and the effect of a gluten-free diet. We focused on vitamins A, E, B9 (folate), B12, and K. The search terms included combinations of “celiac disease”, “deficiency” and the specific vitamins. In addition to PubMed, medical textbooks and other authoritative sources were reviewed to gather further insights on the topic. These sources were chosen for their scientific reliability and relevance to the subject matter.

### 2.2. Inclusion and Exclusion Criteria

Only studies that addressed the association between vitamin deficiencies and celiac disease were included. Articles published in English and focusing on human subjects were considered. Studies not addressing vitamin deficiencies in the context of celiac disease, as well as articles not published in English, were excluded.

### 2.3. Data Collection

In addition to the literature search, a manual review of the references was performed. The selected articles were carefully examined, and the data were extracted based on the reported findings concerning the relationship between celiac disease and vitamin deficiencies. No statistical analysis or data synthesis were conducted, as the primary aim was to summarize and synthesize the findings from the selected studies in a descriptive manner.

### 2.4. Analysis

The articles were analysed qualitatively by reviewing the findings and synthesizing the results related to the impact of celiac disease and a gluten-free diet on vitamin deficiencies. The analysis focused on understanding the prevalence and potential causes of deficiencies in the vitamins of interest, including both the disease itself and the dietary modifications associated with celiac disease.

### 2.5. Discussion

#### 2.5.1. Vitamin A

**Figure 1:**
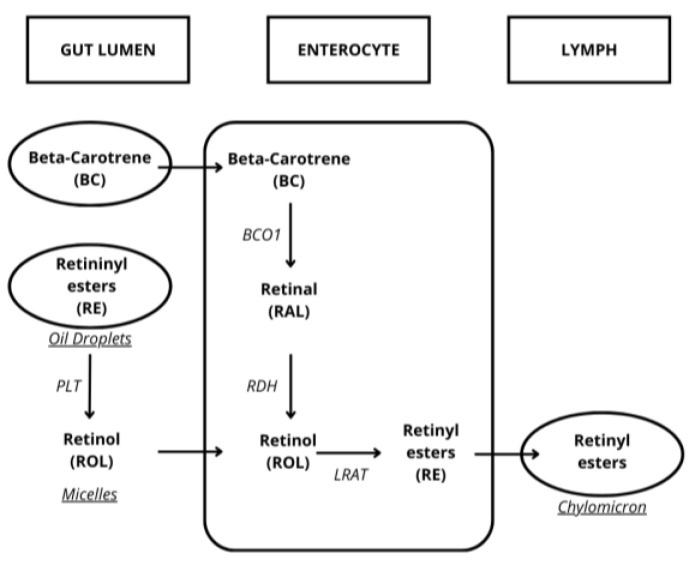
Absorption and transport of vitamin A.

Chesney and Mc Coord described the presence of vitamin A malabsorption in patients with CD in 1934 [19]. Vitamin A is a liposoluble compound, including retinol. Its analogues are known as retinols and are a large group of compounds consisting of up to 1500 different natural and synthetic molecules. Carotenoids are also considered as vitamin A precursors and are subdivided in α-carotene, β-carotene, lutein, zeaxanthin, cryptoxanthin and lycopene. Retinol is the alcoholic form, retinoic aldehyde is the aldehydic form and retinoic acid is the acidic form. They are isoprenoic derivatives formed by four isoprene chains. The absorption of vitamin A, like all liposoluble vitamins, is related to fat absorption. Vitamin A is absorbed in the proximal small intestine as an alcoholic form and then reconverted to an ester before it reaches blood circulation (Figure 1).

Beta-carotene (BC) enters the enterocyte, where it is converted to retinal (RAL) through the action of beta-carotene 15,15′-monooxygenase (BCO1). Retinal (RAL) is then converted to retinol (ROL) by retinol dehydrogenase (RDH). In the enterocyte, retinol (ROL) is esterified to form retinyl esters (REs) through the action of lecithin retinol acyltransferase (LRAT). The retinyl esters (REs) in the gut lumen, in the form of oil droplets, are converted into retinol (ROL) by the action of phospholipid transfer proteins (PLTs). The retinol (ROL) is incorporated into micelles and enters the enterocyte. Once inside the enterocyte, retinol (ROL) is esterified again into retinyl esters (REs) by LRAT. The retinyl esters (REs) are then transported into the lymphatic system in chylomicrons and enter the bloodstream for distribution.

In 1962, in a small group of six symptomatic CD patients, low serum levels of carotene and an insufficient serum increase of vitamin A levels after oral administration of both acetate and alcohol preparations of vitamin A were detected [20]. After the oral administration of 250,000 i.u.% of vitamin A, a peak figure below 800 i.u.% was considered as suggestive of malabsorption. All the patients failed to rise over 600 i.u.%. In two patients, the authors demonstrated the improvement of vitamin A absorption after gluten withdrawal from their diet. In particular, a 50-year-old celiac woman suffering from frank malabsorption symptoms with a long-lasting steatorrhoea, weight loss, hypocalcaemia, abdominal distention and osteomalacia showed a clinical improvement after 6 weeks of a GFD, paralleling an improvement of vitamin A and triolein I^131^ absorption. Moreover, in patients affected by small intestinal disorders other than CD, the authors observed that, although still abnormal, the peaks of vitamin A absorption after oral administration were higher than those detected in CD patients [20]. Vitamin A deficiency is an expected complication in patients with untreated CD with classical manifestation as a consequence of the severe malabsorption.

Vitamin A deficiency is a major cause of ocular morbidity in developing countries, and liver diseases and malabsorption syndromes are the most frequent predisposing conditions in Western countries [21]. However, in patients with CD on a GFD, a severe vitamin A deficiency could also be detected. In a 64-year-old male patient suffering from CD and following an accurate GFD, a severe corneal pathology rapidly healed after vitamin A supplementation was described [22]. The cause of vitamin A deficiency in CD patients on a GFD was clarified by Shepherd and colleagues. They determined the nutritional adequacy of a GFD for 55 patients following a strict GFD for more than 2 years, and 50 newly diagnosed patients prospectively followed a GFD over 12 months [17]. Data were also compared with the general population. The authors observed a similar nutritional intake among the groups, both female and male; one patient out of three had an inadequate mean dietary intake of vitamin A, according to the recommended daily intake [17]. Moreover, in comparison with 24 healthy subjects, Wierdsma and colleagues studied the nutritional status and vitamin A serum levels in 80 newly diagnosed adult CD patients [23]. Vitamin A deficiency was rare in healthy subjects, but in untreated CD, before starting a GFD, low levels of vitamin A were present in a minority of the patients: 7.5% of the cohort studied [23]. Vitamin A deficiency was not associated with the severity of intestinal lesions or the prevalence of obesity or being overweight at diagnosis. Accordingly, extensive nutritional assessments are warranted to guide nutritional advice and follow-up in the treatment of CD [23].

These results were recently confirmed by Unalp-Arida and colleagues in a survey evaluating nutrient intake from the diet among people with CD on a GFD, examining data of the cross-sectional USA National Health and Nutrition Examination Survey from 2009 to 2014 [24]. Data were obtained in patients with CD and subjects without CD diagnosis but spontaneously avoiding gluten. In comparison with CD patients on a GFD, patients without CD but avoiding gluten in their diet and controls of the general population, CD patients at diagnosis, before starting a GFD, showed higher vitamin A intake. The authors concluded that nutriomics studies of multiple analytes measured simultaneously could help to inform screening for malabsorption and treatment strategies [24].

In 182 CD patients included in the registry of patients seen at the adult McMaster Celiac Disease Clinic, 57% were on a long-term GFD, but only 52.4% were strictly adherent to the diet; consequently, 31% had a positive serology and 69.2% had gastrointestinal symptoms. Nevertheless, only 11% of patients had low serum levels of vitamin A, independently from both the length and the level of adherence to a GFD [25]. Vitamin A deficiency seems related more to the adequacy of the diet rather than malabsorption.

In a Norway group of 59 adult female CD patients on a GFD for a period ranging from 1.5 to 44 years, with an excellent (51%) or good (49%) adherence to diet, and both normal histology and serology but persistently suffering from gastrointestinal symptoms, 73% of cases complained regarding the daily requirement for vitamin A. It is therefore conceivable that dietary education and nutritional follow up could be helpful to ascertain whether CD patients follow an unbalanced GFD [26]. In a Spanish study on female patients, four patients out of ten did not fulfil the dietary reference intake for vitamin A and only 11% of patients reached an intake of 2/3 of the reference intake [27]. A similar study of the same group in male patients showed that 81% of patients fulfilled at least 2/3 of recommendation [28].

These results, taken together and summarized in Table 1, suggest that an unbalanced diet, frequently observed in patients following a GFD, is more important than the presence of malabsorption as a risk factor for vitamin A deficiency. Nutritional advice regularly administered in a structured nutritional follow up represent a pivotal strategy in the clinical approach to CD.

#### 2.5.2. Vitamin E

Among the eight compounds known to have the biologic activity of vitamin E, α-tocopherol represents the one that provides the highest activity. Vitamin E is a natural antioxidant comprising two groups of eight isoforms: α-, β-, γ-, and δ-tocopherols and α-, β-, γ-, and δ-tocotrienols. These isoforms or vitamers are differentiated based on the number and position of methyl groups on their common chromanol ring and the presence of a saturated (tocopherol) or unsaturated (tocotrienol) side chain [29].

The main vitamin E sources are vegetable oils; nevertheless, significant quantities of vitamin E isoforms are found in most cereal grains. The potential health benefits of vitamin E include the prevention of heart diseases, several chronic diseases and cancers [29].

The presence of vitamin E deficiency in CD was described in 1946 by WJ Darby and coworkers [30]. In three patients in relapse or early remission, low plasma levels of tocopherol were detected. In one patient with CD, in comparison with a small group of healthy volunteers, the oral administration of tocopherol evoked a lower plasma level increase. Moreover, in a group of CD patients supplemented for a long period, mean plasma levels proved to be normal. These results suggest the role of an impaired intestinal absorption in the pathophysiology of vitamin E deficiency and the normalization with a GFD.

Vitamin E deficiency may be associated to neurological disorders characterised by neuromyopathy, cerebellar ataxia, posterior and lateral column abnormalities, myelopathy or cerebral, brainstem and peripheral nerve involvement [31,32]. In rare patients suffering from CD encephalopathy, epilepsy, cerebellar abnormalities, spinocerebellar degeneration, myelopathy, peripheral neuropathy and psychiatric disturbances were reported [33,34]. In addition, other nutritional deficiencies secondary to nutrient malabsorption may induce neurological disorders and CD may present a neurological involvement from the very beginning or may be complicated by neurological changes during the course of the disease [35].

Several clinical case descriptions have raised the question whether the coexistence of vitamin E deficiency in CD is responsible for these neurological manifestations. In 1985, Ward and colleagues reported on a 47-year-old man with CD and frank malabsorption suffering from spinocerebellar degeneration. Serum vitamin E levels were normal. A GFD and vitamin E supplementation were started; small bowel histology improved, but the neurologic disorder initially deteriorated and later stabilized [36]. Ackerman and colleagues reported on a 45-year-old man with CD with a progressive neurological syndrome and vitamin E deficiency. He suffered from a chronic diarrhoea from the age of 12 and during the last 4 years presented a worsening of this condition associated with a weight loss of 10 kg. CD diagnosis was made based on small bowel histology results, a GFD was started and the regression of diarrhoea and weight gain were obtained. In the following months, a scarce compliance to the GFD caused a relapse of malabsorption, with diarrhoea and weight loss. Quadriparesis occurred together with impairment of proprioception in the lower extremities. Levels of vitamin E were under normal values and failed to rise after oral administration. The patient refused parenteral vitamin E administration; he became demented and was lost at follow-up [37]. Mauro and colleagues reported a case of a woman suffering from adult-onset CD with a cerebellar syndrome with progressive worsening despite the regression of malabsorption symptoms after the beginning of a GFD. The patient showed a vitamin E deficiency and the cerebellar symptoms improved with vitamin E supplementation. This case report supported a possible role of vitamin E deficiency in the development of the neurological complications of CD [38].

Battisti and colleagues described an adult-onset CD patient with severe vitamin E and IgA deficiencies associated with frank malabsorption, severe diarrhoea, steatorrhea and weight loss. Peripheral neuropathy and cerebellar impairment were present. An alteration of nerve conduction velocities, brainstem auditory evoked response and somatosensory evoked potential tests were also present. Radiological studies detected the presence of cortical atrophy in the frontal and parietal regions; nerve biopsy showed a severe nerve fibre loss, skin biopsy showed deposits of lipofuscin in the patient’s skin and duodenal biopsy showed villous atrophy and hyperplasia of the crypts. A GFD and parenteral administration of vitamin E were prescribed, obtaining the normalization of plasma levels of vitamin E within the next 6 months of treatment. The progressive improvement of both clinical and neurological symptoms and disappearance of lipofuscin deposits were obtained [39]. Another adult-onset CD patient, diagnosed at the age of 69 years old, with neurological manifestations such as neuromyopathy, ataxia, and polyneuropathy, proximal weakness, non-length-dependent sensory neuropathy, optic atrophy and a cerebellar syndrome was reported [40]. An history of diarrhoea with hyporexia and 20-kg weight loss present for at least 6 years prior to the neurological manifestations was reported by the patient. Histology and serology were suggestive for CD and a very low level of vitamin E was detected. Muscle biopsy showed the presence of lesions resembling an inclusion-body myositis, with inflammatory infiltrates and rimmed vacuoles. The starting of a GFD associated to vitamin E, folic acid and vitamin D supplementation induced the regression of diarrhoea, the improvement of appetite and weight and the concomitant improvement of cerebellar manifestations. Post-treatment muscle histology 1 year after starting a GFD showed a marked improvement of the lesions. In this patient, a GFD with vitamin E supplementation reverted both the neurological manifestations and proximal weakness [40]. Henri-Bhargava and colleagues described a 58-year-old man with mild, longstanding CD and dermatitis herpetiformis who complained of leg stiffness and gait unsteadiness [41]. During the last decade, replacement therapy due to iron-deficiency anaemia and vitamin B12 deficiency was administered, but he showed a very low compliance to a GFD. Cerebellar degeneration and myeloneuropathy due to vitamin E and copper deficiencies due to prolonged micronutrient malabsorption secondary to the lack of GFD compliance were evident. A rigorous GFD associated with vitamin E and copper supplementation were prescribed, and a moderate improvement in cerebellar function and gait after 12 months was detected [41].

The clinical overlap between neurological manifestation of vitamin E deficiency and neurological manifestations associated to CD evoked the suggestion of the pathophysiological role of vitamin E deficiency in this condition. Some observations rule out the responsibility of vitamin E deficiency. In CD, neurological manifestations were described only in adults, while in other conditions characterised by fat malabsorption, neurological manifestations appear also in paediatric patients [42]. Low levels of vitamin E at diagnosis normalize without supplementation with the beginning of a GFD [36]. In the series of patients described by Cook and Smith [34], the length of time between the onset of CD and neurological manifestations was 25 years, and most of the patients were diagnosed before the relationship between gluten and CD was ascertained. Consequently, these patients were exposed to the effect of gluten and the malabsorption syndrome for a very long period. It is conceivable that patients suffering from less severe malabsorption could be less exposed to the risk of neurological disorders. However, considering all the information provided by previous clinical cases, the association between vitamin E deficiency and neurological disorders remains uncertain.

Few systematic studies analysed vitamin E levels in CD. In comparison with a group of untreated patients, Hozyasz and colleagues measured both plasma and erythrocyte vitamin E levels in patients on GFD. In untreated patients, studied at diagnosis, vitamin E levels both in plasma and erythrocytes were lower compared to the group of coeliac patients on a GFD. Levels of vitamin E in erythrocytes were below the low normal limit in all the patients with active CD. The author suggested the determination of vitamin E levels as a biomarker for the monitoring of GFD adherence in patients with CD [43]. In a study dealing with oxidative stress, plasma levels of vitamin E were measured in 53 patients with untreated active CD, 92 patients on a GFD for at least 2 years, and 52 control subjects suffering from functional disorders. Celiac patients showed non-classic signs: chronic abdominal pain without typical malabsorption syndrome, osteoporosis, osteopenia and extraintestinal manifestations such as anaemia (iron deficiency) without gastrointestinal symptoms, as well as asymptomatic disease. Untreated and treated patients showed lower serum vitamin E levels than in control subjects. Vitamin E deficiency (serum level under 16.2 μmol/L) was observed in 3.7% of control subjects and in over 60% of celiac patients. Optimal vitamin E levels (>30 μmol/L) required for protection against cancer and cardiovascular disease were observed in more than 96% of control subjects and in less than 40% of celiac patients [44].

In a Canadian study, a cohort of CD patients on a GFD underwent a determination of plasma vitamin E. None of the enrolled patients showed low levels of vitamin E, independently from the duration of the diet, suggesting that a balanced GFD is the main factor preventing micronutrient deficiency in these patients [25].

An interesting point is represented by the nutritional adequacy of a GFD. Studies performed in different countries show different results.

In a Spanish group of 54 adult women on a GFD, an evaluation of the nutritional adequacy of diet was performed. Eight patients out of ten did not fulfil the dietary reference intake and only four out of ten reached the value of 2/3 of the dietary daily recommendation [27]. Similarly, in Spanish CD male patients, it was shown that 48% of participants fulfilled at least 67% of the recommendation [28]. Another Spanish study on CD patients on a GFD substantially confirmed these results, showing that recommended daily vitamin E intake was fulfilled by 8 male patients and 6.5 women out of 10 [45]. A US study showed that CD patients on a GFD had higher intake of vitamin E in comparison with controls but mean vitamin E daily intake proved to be clearly below the recommended dietary allowance of 15 mg/day [24]. On the contrary, a Norwegian study showed a 97% of women complying the Nordic Nutrition Recommendation for vitamin E daily intake.

These large differences among studies performed in different countries, and summarized in Table 2, could reflect the availability of food containing different amounts of vitamin E. However, this point seems to be not the case as it was recently shown that vitamin E content in gluten-free foods is similar to the content of the correspondent gluten-containing food [45]. It is therefore conceivable that the main risk factor for a persistent or a new-onset vitamin E deficiency in patients on a GFD is represented by a patient’s food choice.

#### 2.5.3. Vitamin K

**Figure 2:**
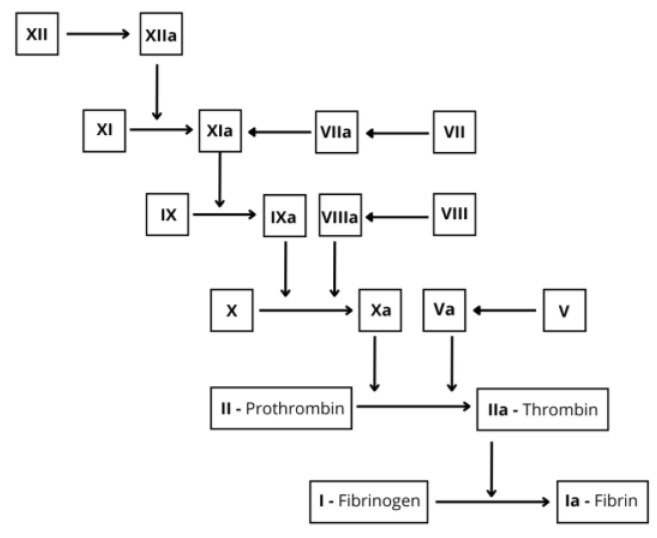
Coagulation cascade and vitamin K-dependent coagulation factors.

Vitamin K is a fat-soluble vitamin available in two forms. Phylloquinoine (vitamin K1) is present in vegetables, and it is absorbed in the small intestine. Menaquinone (vitamin K2) derives from intestinal microbiota metabolism and is produced in the colon [46]. Vitamin K is involved in the synthesis of coagulation factors II, VII, IX and X and a reduction of intestinal absorption causes vitamin K deficiency (Figure 2).

Activation of coagulation factor XII (XII) into activated factor XII (XIIa) leads to the activation of factor XI (XI) into activated factor XI (XIa). Activated factor XIa then activates factor IX (IX) into activated factor IX (IXa). Activated factor IX (IXa), in combination with activated factor VIII (VIIIa) (which was previously factor VIII), activates factor X (X) into activated factor X (Xa). Factor VII (VII) is converted to activated factor VII (VIIa), playing a key role in this pathway. Activated factor X (Xa) combines with activated factor V (Va) (which was previously factor V) to form the prothrombinase complex, which converts prothrombin (II) into thrombin (IIa). Thrombin then cleaves fibrinogen (I) into fibrin (Ia), which polymerizes to form the fibrin clot. Note: factor V (V), factor VII (VII) and factor VIII (VIII) refer to their inactive forms, which are subsequently activated to activated factor V (Va), activated factor VII (VIIa) and activated factor VIII (VIIIa), respectively.

An alteration of coagulative balance may be present in malabsorption syndromes [47,48], and this complication was also described in patients with CD at diagnosis [49,50,51]. However, the alteration of prothrombin time rarely represents a cause of acute bleeding in untreated CD patients [49,52], despite the fact that a considerable modification of prothrombin time was detected in 18.5–25% of untreated CD patients in both a retrospective [53] and a prospective [54] evaluation.

Symptoms or signs caused by coagulopathy, such as hematomas, bruises or frank haemorrhagic manifestations may represent the primary presenting features of undiagnosed CD associated to a malabsorption of vitamin K. However, the spectrum of clinical presentation may be very wide, ranging from an acute disorder with bleeding per rectum associated with occasional diarrhoea [49] to generalized bruises associated to severe diarrhoea [55,56] and severe coagulopathy [57,58]. Mild gastrointestinal symptoms associated with an impaired nutritional status and large haematomas and swellings [59] or diffuse ecchymosis without abdominal pain or diarrhoea [60] were also described. A history of easy bruising following minimal trauma and diffuse hematomas together with weight loss, fatigue, steatorrhea and malabsorption associated with severe anaemia were also described [61]. Combined vitamin deficiency may be present both in patients with [62] and without an overt malabsorption [59] also involving vitamin A, E and D.

The beginning of a GFD allows a progressive but rapid reversal of vitamin K deficiency [54]. In a Spanish study enrolling adult patients on a long-term GFD, it was shown that CD patients respected the recommended dietary intake for vitamin K, without differences between males and females [45], suggesting that an exogenous supplementation is rarely needed.

Table 3 summarizes the results of the studied on vitamin K in CD patients.

It should be also noted that CD is characterized by a hypercoagulability state, as is expected in all the autoimmune disorders [63]. Stroke is reported in both children and adults suffering from CD [64,65], but many other conditions may occur, such as anticardiolipin syndrome, pregnancy loss, peripheral deep vein thrombosis, cardiovascular diseases, small bowel infarction secondary to vascular thrombosis, pulmonary thromboembolism, Budd–Chiari Syndrome, atrial fibrillation and dilated cardiomyopathy [63]. Moreover, the first manifestation responsible for the recognition of the disease may be represented by a thrombotic event [66,67,68]. The frequent finding of hyperhomocysteinemia, in particular in patients at diagnosis, is associated with vitamin deficiency [68,69,70], methylentetrahydrofolate reductase mutations [71,72,73] and the homology between tissue transglutaminase and factor VIII [73], representing other factors predisposing CD patients to a hypercoagulability status.

#### 2.5.4. Vitamin B12

Cobalamin or vitamin B12 is a water-soluble compound with a complex structure. Superior animals are not able to synthesize vitamin B12, as they do not carry genes encoding for cobalamin. On the contrary, bacteria, yeasts and some algae can synthesize vitamin B12. Notably, some of these bacteria colonize the upper gastrointestinal tract of herbivores, explaining why herbivores do not show vitamin B12 deficiency despite an insignificant dietary intake. Other than Asiatic mushrooms, some algae and some yeast products, vitamin B12 is present in foods of animal origins: liver, meat, kidney, milk, eggs, fish and shellfish [74]. The deficiency of vitamin B12 predisposes patients to thromboembolic consequences as it worsens the haemocoagulative balance.

**Figure 3:**
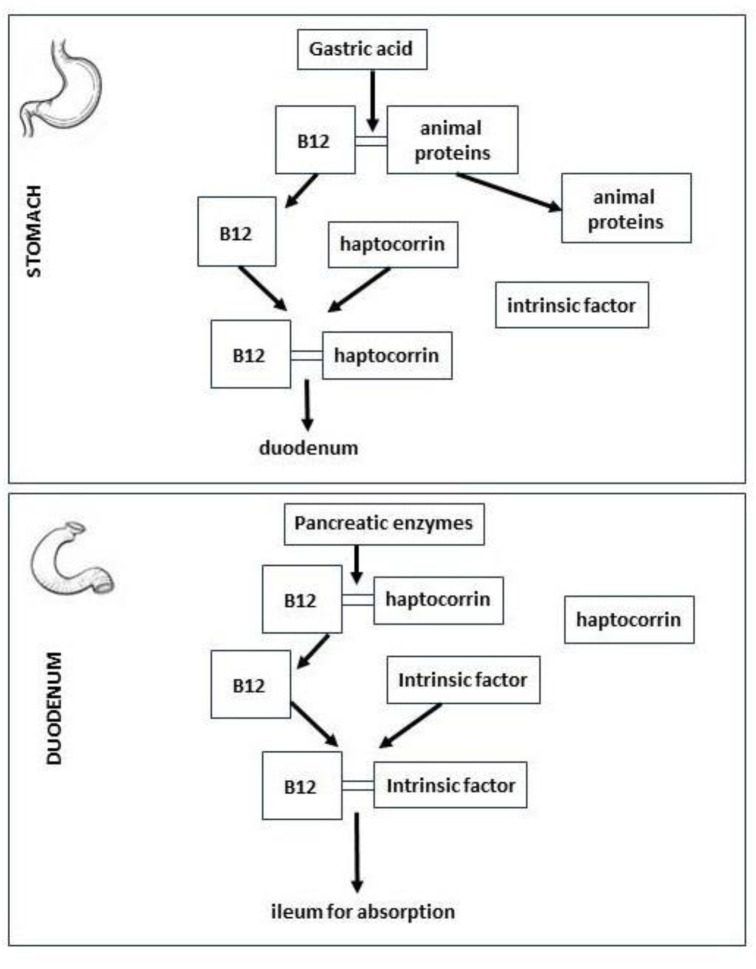
Absorption of vitamin B12.

Vitamin B12 is released from food proteins and binds endogenous carriers in the stomach. Haptocorrin, intrinsic factor and transcobalamin proteins and their membrane receptors are involved in the complex mechanism of absorption and transport of vitamin B12. Haptocorrin derives from saliva and binds 10–40% of cobalamin in the gastric acid juice. The low gastric pH enhances the affinity of haptocorrin for vitamin B12 rather than for the intrinsic factor. Then, the pancreatic enzymes metabolize haptocorrin, and in the duodenum vitamin B12 is transferred to the intrinsic factor. In the ileum, the cobalamin-intrinsic factor complex consents the absorption of more than 98% of vitamin B12 by receptor-mediated endocytosis. However, a small amount is absorbed along the entire small bowel too [75]. Finally, transcobalamin carries cobalamin to the liver, where it is stored, but enterohepatic circulation is also present [76] (Figure 3).

Haptocorrin is the Vitamin B12 carrier in the stomach and intrinsic factor is the carrier from the duodenum to the ileum.

Several studies reported that serum levels of vitamin B12 were insufficient in up to 41% of CD patients at diagnosis [77,78,79,80,81]. Nevertheless, the prevalence of vitamin B12 deficiency in the majority of these studies ranged from 5 to 12% [77,78,80,81]. It has been found to be very high only in one Scottish paper showing vitamin B12 deficiency in 41% of cases [79]. The main cause for this discrepancy was considered a severe clinical presentation without autoimmune gastritis. Nonetheless, weight loss, abdominal symptoms and stool characteristics were not predictive of vitamin B12 deficiency [81]. The clinical guidelines underline the high frequency of micronutrient deficiencies in CD at the time of diagnosis [82] and that nutritional supplementation is required mainly in the early stages after diagnosis [83].

Micronutrient deficiency may persist in CD patients who strictly follow a long-term GFD. In spite of a strict GFD for an average period of 16 months, a complete reversal of mucosal lesions occurred in only 8% of CD patients diagnosed in adulthood.

Furthermore, in 65% of CD patients, the remission was linked to intraepithelial lymphocytosis, and in 27% of CD patients there was a lack of histological response [84]. Although this kind of slow response is frequent in adult CD patients over 50 years of age [85], this slow response may happen also in one out of five children after 1 year of a GFD [86].

Nonetheless, a persisting deficiency of vitamin B12 is uncommon in CD patients on a strict GFD. In 30 adults CD patients on a long-term GFD (8 to 12 years), none of them presented vitamin B12 deficiency and all patients showed high vitamin B12 intake [69]. Supplementation increased serum levels of vitamin B12 and an improvement of clinical symptoms in CD patients was also observed, suggesting that the definition of the lower limit of normality was rather unclear [70]. An adequate intake of vitamin B12 was observed in all enrolled patients on a GFD in three Spanish studies [27,28,45] and one Canadian study [25]. In the Canadian study, only one patient on a GFD for a period of less than 24 months showed low vitamin B12 serum levels. Likewise, in a study from Norway, only one CD patient with persistent gastrointestinal symptoms showed an insufficient vitamin B12 intake [26]. The low prevalence of vitamin B12 deficiency in CD patients following a GFD could be explained by the higher vitamin B12 content of gluten-free breads, corn flakes and pasta in comparison with the corresponding gluten-containing foods [74]. Furthermore, vitamin B12 intestinal absorption through a passive route along the small bowel may enable the restoration and maintenance of its normal serum levels [85].

Hence, when there is a vitamin B12 deficiency in CD patients on a rigorous GFD, additional examinations are mandatory to exclude an accidental gluten intake and the coexistence of other undiagnosed disorders, such as autoimmune atrophic gastritis that potentially causes impaired vitamin absorption [87].

**Figure 4:**
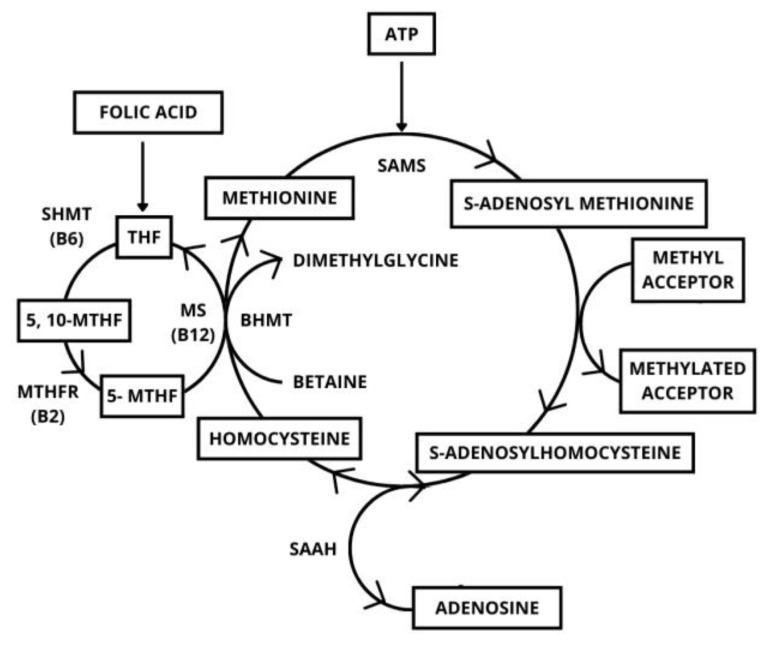
Metabolic cycle of adenosine.

In untreated adult CD patients, increased levels of homocysteinemia rapidly improved after some months of a GFD [88]. The deficiencies of vitamin B9 and vitamin B12 are both implicated in hyperhomocysteinemia and suggest the pivotal role of these two deficiencies in the pathogenesis of vascular complications linked to disorders of the homocysteine metabolic pathway (Figure 4).

Methionine is transformed into homocysteine through the activity of S-adenosylmethionine synthetase (SAMS), methyltransferase (MT) and S-adenosylhomocysteine hydrolase (SAHH) acting in sequence. Then, homocysteine is remethylated to methionine by methionine synthase (MS); in presence of folate and vitamin B12 and betaine homocysteine S-methyltransferase (BHMT), in presence of a metabolite of choline, betaine. To remethylate homocysteine via MS, 5-methyltetrahydrofolate (5-MTHF) is needed, which is derived from 5,10-methylenetetrahydrofolate (5,10-MTHF) in a reaction catalysed by methylenetetrahydrofolate reductase (MTHFR) with vitamin B2 as a cofactor. Finally, 5-MTHF is transformed in tetrahydrofolate (THF), which in turn is transformed into 5,10-MTHF by serine hydroxymethyltransferase (SHMT), and vitamin B6 represents the cofactor to complete the folate cycle.

Both vitamin B9 and vitamin B12 are required in the catabolism of homocysteine; therefore, hyperhomocysteinemia may reveal a deficiency of both vitamins. In adult CD patients, a low vitamin B9 intake should be considered responsible for steady hyperhomocysteine [17,45,81,82], even in CD patients on a long term GFD [24,25,26,27,28]. It should also be highlighted that gluten-free foods contain lower quantities of vitamin B9 compared to their gluten-containing equivalents [89].

Table 4 summarizes the main studies on vitamin B12 in CD patients.

#### 2.5.5. Folate/Vitamin B9

The term “folate” refers to a group of water-soluble B-vitamin compounds, primarily known as vitamin B9, that humans cannot synthesize. It exists in multiple forms, including naturally occurring polyglutamates found in foods and the synthetic monoglutamate form, folic acid, commonly used in dietary supplements and fortified foods. Naturally occurring food folate is absorbed mainly in the duodenum and upper jejunum of the small intestine. Before absorption, it is converted to monoglutamates by the intestinal enzyme folylpoly-γ-glutamate carboxypeptidase, enabling active transport across the intestinal mucosa. In contrast, folic acid, already in the monoglutamate form, bypasses this step and is absorbed via passive diffusion. Once inside enterocytes, folate is reduced and methylated to form 5-methyltetrahydrofolate (5-MTHF) that then enters systemic circulation after being transported to the liver. Folate plays a critical role as a coenzyme or cosubstrate in single-carbon transfer reactions essential for nucleic acid synthesis (DNA and RNA) and amino acid metabolism. One key folate-dependent reaction is the conversion of homocysteine to methionine, which is vital for the synthesis of S-adenosyl-methionine, an important methyl donor. Another important reaction involving folate is the methylation of deoxyuridylate to thymidylate, which is necessary for DNA formation and proper cell division. Disruptions in these processes can lead to megaloblastic anaemia, which is a well-known consequence of folate deficiency [90,91,92,93,94].

Folate also works with vitamin B12 in red and white blood cell production, as well as in preventing neural tube defects (NTDs) in developing foetuses. Adequate folic acid intake is essential for normal haematopoiesis and proper nervous system development [95,96]. Given its essential roles in the body, obtaining sufficient folate from the diet is crucial. Folate is naturally present in a wide variety of foods, including vegetables (especially dark green leafy vegetables), fruits and fruit juices, nuts, beans, peas, seafood, eggs, dairy products, meat, poultry and grains. Foods high in folate include spinach, liver, asparagus and brussels sprouts [93,97].

While adequate folate intake is essential, deficiency can lead to significant health issues. The primary clinical manifestation of folate deficiency is megaloblastic anaemia, which is characterized by large, abnormally nucleated erythrocytes [90,91,92,93]. Symptoms include weakness, fatigue, difficulty concentrating, irritability, headache, heart palpitations and shortness of breath [91]. Additionally, folate deficiency may cause soreness and shallow ulcers on the tongue and oral mucosa; alterations in skin, hair, or fingernail pigmentation; gastrointestinal issues; and elevated blood levels of homocysteine [90,91,92,93]. Women with insufficient folate intakes are at increased risk of giving birth to infants with NTDs [91]. Insufficient maternal folate status is also linked to low birth weight, preterm delivery and foetal growth retardation [90,98]. In patients with CD, folate deficiency can lead to macrocytic and megaloblastic anaemia, along with abnormalities in other blood cell lines [99]. Severe folate deficiency may result in reduced leukocyte and platelet counts, potentially manifesting as severe pancytopenia [99]. Untreated CD has been found to be a major contributor to this form of macrocytic anaemia. In a large European study, it has been shown that up to 20–34% of untreated CD patients present with anaemia, largely due to malabsorption [78].

Table 5 summarizes the results of studies on vitamin B9 in CD patients.

The first significant study on this topic reported high rates of folate deficiency in newly diagnosed CD patients: a study from the 1990s found that 81% of a cohort of 16 children diagnosed with CD had low serum folate levels [100]. These results were confirmed in adults by Hallert et al. in 1998, showing that decreased serum folate concentration is a frequent abnormality in a cohort of CD patients [101]. Haapalahti et al. studied the nutritional status of 26 adolescent CD patients, finding that one-third had low folate levels at the time of diagnosis [102]. Other research reinforced these findings, showing folic acid deficiency was observed in 20% of the 80 untreated CD patients [23]. The prevalence of folate deficiency varies from 18% to 90% in both old and recent reports on CD patients [101,103,104]. A study from Finland, for instance, reported folate deficiency in 37% of 40 untreated CD patients. In untreated CD patients, a higher severity of villous atrophy was associated with a lower folate and higher homocysteine levels [105]. It is conceivable that proximal villous atrophy may account for micronutrient malabsorption in untreated patients and the higher the degree of villous atrophy, the more severe the folate deficiency [105]. A strict adherence to a GFD leads to the improvement of mucosal architecture, in turn improving nutrient absorption. McFarlane et al. showed that 55 adult CD patients on a GFD had red cell folate concentrations within the normal range [106]. Notably, other studies demonstrated that a GFD normalizes folate status and reduces plasma homocysteine levels [72,105]. However, it was also reported that there is a persistent reduction of folate levels on a GFD [107,108]. Moreover, Dickey et al. did not observe any difference of serum folate concentrations among patients with sufficient and incomplete recovery of mucosal architecture and controls [72]. Hallert et al. showed that 20% of a cohort of adult CD patients on a 10-year period of a GFD, despite having a normalized intestinal mucosa, still exhibited folate deficiency. They suggested that this could be attributed to other factors, such as inadequate nutrient consumption [69]. Accordingly, if a GFD helps, it may not completely resolve folate deficiency in all patients. Altered folate status may reflect the shortcomings of a GFD, which has been shown to be deficient in various nutrients [109]. Indeed, lower daily folate intake has been reported in treated CD patients compared to controls [69,109,110]. This observation aligns with evidence that gluten-free cereal products, such as breads and pastas, contain lower amounts of folate compared to their gluten-containing counterparts [89].

Other than anaemia, long-term folate deficiency may lead to elevated serum concentrations of homocysteine, which increase the risk of developing coronary conditions [98,111]. While studies have noted hyperhomocysteinemia in a significant proportion of untreated CD patients, this risk appears to diminish with long-term adherence to a GFD. However, cases of hyperhomocysteinemia were described in treated patients, suggesting that inadequate folate intake from a GFD might still pose a risk for cardiovascular complications. Zanini et al. found that in untreated CD, the prevalence of hyperhomocysteinemia was 46% among 67 patients compared to healthy controls; this percentage decreased to 24% after 5 years of treatment with a GFD [107]. In treated patients, studies evaluating this prevalence remain limited. Notably, Hallert et al. found that hyperhomocysteinemia can persist even after 10 years of adherence to a GFD [69]. Furthermore, Dickey et al. reported a significant decrease in serum homocysteine after 1 year of a GFD, while De Marchi et al. did not observe this effect after 6–8 months of treatment [72,112]. Therefore, more studies are needed to clarify the relationship between adherence to a GFD and serum homocysteine concentrations. Additionally, CD patients have an increased risk of venous thromboembolism (VTE) and vascular diseases but the mechanisms responsible for such extra-intestinal complications are not known [113,114]. Folate deficiency and hyperhomocysteinemia may be involved as risk factors for these complications [107].

Despite the importance of a GFD on a CD patient’s health, few studies assessed the nutritional adequacy of this diet. In comparison with a control group, treated CD patients showed a lower consumption of vitamin B1, B2, B6 and folate intake [17,109,110]. However, to the best of our knowledge, only two studies compared the vitamin intake adequacy in relation to nutritional recommendations, showing the real supply of nutrients by a GFD [16,115]. Gluten-free cereal products generally provide lower amounts of folate than their gluten-containing counterparts, causing a lower intake of these nutrients by CD patients on a GFD due to their production with refined flours without any fortification [89]. Moreover, the inadequacy of the habits and food choices of CD patients may increase the risk of nutritional deficiencies [69,116]. Lee et al. showed that CD patients did not reach the minimum recommendation of six servings of whole grains a day, needed to achieve an adequate daily folate intake [117]. In the general population, enriched fortified cereal products contribute a large percentage to the daily intake of folate. Based on data from the US Department of Agriculture 1989–91 Continuing Survey of Food Intakes by Individuals, ready-to-eat cereal and yeast bread contribute 28.3% of the US adult daily intake of folate [118]. As a result, CD patients following a GFD may have lower overall intake of folate, exacerbating the risk of deficiency. Therefore, it cannot be concluded that adherence to a GFD necessarily leads to an inadequate folate intake [16]. A GFD is simple in its principles; however, to completely eliminate all foods and ingredients that contain gluten is a task that requires a lot of effort and commitment. Health professionals have the role of guiding the patients so that a GFD could be healthy, interesting and practical [119]. These goals are difficult to be achieved for patients who are not professionally oriented because the diet imposed is restrictive, and the changes required are difficult and permanent [120]. Accordingly, more attention should be given to the quality of the nutrients offered by a GFD because this constitutes a life-long treatment [108]. It is recommended folate supplementation associated with a GFD for the treatment of these patients [121].

**Table 5:** Summary of key findings on folate deficiency and nutritional status in CD. Abbreviations: CD: celiac disease, GFD: gluten-free diet, BMD: bone mineral density, HDL: high-density lipoprotein and BMI: body mass index.

Authors and Year	Study Objective	Population	Key Results	Conclusions
Hallert et al., 1981 [101]	Serum folate as a screening test for adult CD	48 untreated adult CD patients (30 female, 18 male patients) from a gastroenterology clinic	85% had low serum folate	Folate is a reliable screening test for CD
McFarlane et al., 2001 [106]	BMD in treated adult CD patients	45 female and 10 male patients with adult-diagnosed CD on a GFD diet	50% of males and 47% of females had osteoporosis; lower BMI and calcium intake correlated with low BMD	GFD helps prevent bone loss in early stages of CD
Dickey et al., 2002 [80]	Prevalence of low serum vitamin B12 in CD	159 CD patients (13 with low B12 at diagnosis, with 6 on B12 therapy)	12% prevalence of low B12 that was unrelated to clinical characteristics	Low B12 is common in CD and is unrelated to autoimmune gastritis
Hallert et al., 2002 [69]	Vitamin status in CD patients on a long-term GFD	30 adults with CD (mean age 55 years, 60% females) in biopsy-proven remission	High homocysteine levels; low folate in 37% and low vitamin B6 in 20% of patients	Vitamin status in CD patients on a long-term GFD should be monitored
Haapalahti et al., 2005 [102]	Nutritional status in newly diagnosed CD patients	26 CD patients (16–25 years) and 29 healthy controls (16–21 years)	Low folic acid, ferritin, pre-albumin; high transferrin receptor levels; 31% of CD patients had subnormal folic acid	Early diagnosis and diet change are key factors for addressing deficiencies
Lee et al., 2009 [117]	Improve the GFD with alternative grains	Retrospective review by a CD specialist dietitian	Substitution with oats, quinoa and high-fibre bread improved protein, iron, calcium and fibre intake	Alternative grains enhance the nutritional profile of a GFD
De Marchi et al., 2013 [112]	Early atherosclerosis signs in young CD adults	20 adults at first diagnosis of CD, after 6–8 months of a GFD, and 22 healthy controls	Increased carotid intima–media thickness; improved cholesterol and HDL after a GFD	A GFD improves vascular health, but CD patients may still be at risk
Zanini et al., 2013 [107]	Impact of a GFD on cardiovascular risk	715 CD patients; retrospective analysis of 1–5 years of a GFD	Increased BMI, cholesterol and γ-glutamyl transpeptidase; decreased triglycerides and homocysteine	A GFD affects risk factors, but it is not conclusively atherogenic

## 3. Conclusions

Vitamin deficiency in celiac disease is an important clinical problem even in the absence of frank malabsorption. The improvement of the knowledge on both pathophysiology and clinical aspects of this condition, in particular in the last twenty years, has allowed physicians to reduce diagnostic delay and recognize CD patients in an early phase of the disease, which is usually characterized by a mild clinical presentation and rarely associated to severe complications. Accordingly, the burden of the disease caused by vitamin deficiency is radically changed in countries where its prevalence is high: clinical consequences of vitamin A and E deficiencies are now very rare, but clinical consequences of vitamin B12 and folate deficiencies, eventually associated with vitamin K deficiency, should be still taken into account, with a great attention, due to the severity of complications determined by these deficiencies. Neurological manifestations, thromboembolic conditions and bone marrow hypofunction still affect the disease course of many patients. Consequently, a strong suggestion arises from available data, supporting the revision of clinical guidelines to formulate practical recommendations considering type and timing of diagnostic tests to drive an early diagnosis of vitamin deficiencies and a correct follow up to prevent specific complications.

Finally, particular attention should be paid to identify vitamin deficiencies related to an incorrect diet. A GFD is the treatment of this condition but the choice of foods is pivotal to guarantee a balanced nutrient intake. The adoption of an incorrect dietary regimen, reducing the variability of the diet by avoiding systematically some foods, may be responsible for vitamin deficiency despite a strict GFD. Consequently, the importance of nutritional advice for CD patients strongly emerges; dietary and nutritional surveillance, organized according to a specific follow up, are an essential to allow a balanced nutrient intake and prevent nutritional deficiencies.

## Figures and Tables

**Table 1:** Summary of key findings on nutritional deficiencies and nutrient intake in CD related to vitamin A. Abbreviations: CD: celiac disease and GFD: gluten-free diet.

Authors and Year	Study Objective	Population	Key Results	Conclusions
Wierdsma et al., 2013 [23]	Assess vitamin/mineral deficiencies in newly diagnosed CD	80 CD patients and 24 controls	87% had deficiencies; common in vitamin A, B6, B12 and zinc	Deficiencies are common in newly diagnosed CD, regardless of intestinal damage severity
Unalp-Arida et al., 2022 [24]	Compare nutrient intake in CD with a GFD	15,610 participants including CD patients, PWAG and controls	CD patients had higher vitamin A intake	Nutrient intake differs significantly between treated and untreated CD, requiring further research

**Table 2:** Summary of key findings on vitamin E deficiency and its neurological effects in CD. Abbreviations: CD: celiac disease and GFD: gluten-free diet.

Authors and Year	Study Objective	Population	Key Results	Conclusions
Mauro et al., 1991 [38]	Investigate vitamin E deficiency and cerebellar syndrome in CD	A 47-year-old female CD patient	Cerebellar symptoms improved with vitamin E therapy	Vitamin E deficiency may cause neurological complications in CD
Hozyasz et al., 2003 [43]	Investigate vitamin E status in CD patients	30 CD patients (range: 2–53 years)	Untreated CD patients had lower tocopherol levels compared to those on a GFD	A GFD improves vitamin E status, but monitoring tocopherol levels may benefit non-compliant or new patients
Kleopa et al., 2005 [40]	Examine myopathy with vitamin E deficiency in CD	A 69-year-old male CD patient	Myopathy improved with vitamin E and a GFD	Vitamin E deficiency can cause reversible myopathy in CD
Henri-Bhargava et al., 2008 [41]	Investigate vitamin E-related neurologic impairment and copper deficiency	A 58-year-old male CD patient	Neurologic symptoms improved with vitamin E and copper supplementation along with a GFD	Untreated CD can lead to neurologic complications that are reversible with appropriate therapy

**Table 3:** Summary of key findings on vitamin K deficiency in CD. Abbreviations CD: celiac disease, GFD: gluten-free diet and INR: international normalized ratio.

Authors and Year	Study Objective	Population	Key Results	Conclusions
Graham et al., 1982 [49]	Report CD as acute bleeding disorder	2 CD young female patients with acute bleeding	Severe bruising, abnormal prothrombin time and malabsorption	Vitamin K and a GFD improve bleeding symptoms
Hussaini et al., 1999 [55]	Report on vitamin deficiencies in untreated CD	A 32-year-old female patient with untreated CD	Vitamin K, A and E deficiencies and elevated prothrombin	Vitamin K deficiency responds to a GFD
Cavallaro et al., 2004 [53]	Prevalence of prolonged prothrombin time in untreated CD	390 adults with untreated CD	18.5% had prolonged prothrombin time and lower haemoglobin, iron, and cholesterol; 5.6% needed vitamin K	Prolonged prothrombin time is linked to severe malabsorption; no need to screen subclinical CD for coagulation disorders
McNicholas & Bell, 2010 [56]	CD causing hypocalcaemia, osteomalacia and coagulopathy	A 36 year-old male CD patient	Symptoms: muscle weakness, osteomalacia, low calcium, vitamin D deficiency and INR 2.7	CD can present with metabolic bone disease and coagulopathy; treated with calcium and vitamin K

**Table 4:** Summary of key findings on vitamin B12 deficiency in celiac disease. Abbreviations: CD: celiac disease, GFD: gluten-free diet, VA: villous atrophy, IgA tTG: immunoglobulin A tissue transglutaminase and tTG: tissue transglutaminase.

Authors and Year	Study Objective	Population	Key Results	Conclusions
Bodø & Gudmand-Høyer et al., 1996 [77]	Symptoms, diagnostic delay and haematologic features in CD	50 adult CD patients	Tiredness (78%), borborygmus (72%), abdominal pain (64%), diarrhoea (56%), weight loss (44%), anaemia (22%) and liver involvement (19%)	CD has subtle clinical features in adults: low haematologic abnormalities
Dahele & Ghosh et al., 2001 [79]	Prevalence of vitamin B12 deficiency in untreated CD	39 CD patients: 32 female and 7 male; median age 48 years (range: 22–77 years)	41% of patients had B12 deficiency; anaemia normalizes on a GFD	Vitamin B12 deficiency is common in untreated CD and requires supplementation
Dickey et al., 2002 [80]	Prevalence of low serum vitamin B12 in CD	159 CD patients (13 with low B12 at diagnosis, with 6 on B12 therapy)	12% prevalence of low B12; no link with clinical characteristics	Low B12 levels are common in CD and are related to autoimmune gastritis
Harper et al., 2007 [78]	Causes of anaemia in CD	405 CD patients	iron deficiency in 33% of men, 19% of women; 12% with folate and 5% with vitamin B12 deficiencies; 20% with anaemia; a GFD affects ferritin	Anaemia in celiac disease is multifactorial: both nutritional deficiencies and inflammation contribute
Lanzini et al., 2009 [84]	Effect of a GFD on mucosal recovery	465 adult CD patients during a GFD (gender and age not specified)	8% histological normalization, 65% remission, 26% no change; 83% Marsh III had negative serology	Complete mucosal recovery is rare despite optimal adherence to a GFD
Lebwohl et al., 2014 [85]	Predictors of persistent villous atrophy in CD	7648 CD patients (age range not specified)	43% had persistent VA; increased age and male gender linked to higher prevalence	Persistent villous atrophy linked to age and gender; effect of GFD on mucosal recovery
Leonard et al., 2017 [86]	IgA tTG and mucosal recovery in CD in children on a GFD	103 paediatric CD patients (<21 years)	19% persistent enteropathy; tTG predictive value: 25% (positive), 83% (negative).	tTG levels are an unreliable marker for mucosal recovery detection; symptoms and serology not predictive
Bledsoe et al., 2019 [81]	Micronutrient deficiencies in newly diagnosed CD	309 newly diagnosed CD patients (196 women and 113 men; mean age 46.1 years)	Patients with low zinc (59.4%), albumin (19.7%), copper (6.4%), vitamin B12 (5.3%), folate (3.6%), vitamin D (19%) and ferritin (30.8%)	Micronutrient deficiencies are present even without overt malabsorption

## Data Availability

Not applicable.
